# Response to Commentary: Hand and Grasp Selection in a Preferential Reaching Task: The Effects of Object Location, Orientation, and Task Intention

**DOI:** 10.3389/fpsyg.2018.00905

**Published:** 2018-06-05

**Authors:** Sara M. Scharoun Benson, Pamela J. Bryden

**Affiliations:** ^1^Department of Kinesiology, University of Windsor, Windsor, ON, Canada; ^2^Department of Kinesiology and Physical Education, Wilfrid Laurier University, Waterloo, ON, Canada

**Keywords:** preferential reaching, hand selection, joint action, grasp selection, object orientation, task intention, Object location

We agree with Moreau and Candidi (2016) that the definition of “joint action” should be carefully considered. A review of articles within the psychology and motor neuroscience area utilizing the term “joint action” reveals a myriad of uses of the term, along with a plethora of experimental techniques to examine the concept.

Most researchers in the last decade utilize the Sebanz et al. (2006) definition of joint action. Here, they state that “joint action can be regarded as any form of social interaction whereby two or more individuals coordinate their actions in space and time to bring about change in the environment” (p. 70). Such a definition is inclusive of many actions, including the actions of birds flocking, argues Milward and Carpenter (2018), and the picking up and passing of objects between individuals (Schmitz, 2017). Other examples of joint action include “doing the dishes together, rowing a canoe together, or playing a piano duet” (Knoblich and Sebanz, 2006, p. 100). Sebanz et al. (2006) further argued that “successful joint action depends on the abilities (i) to share representations, (ii) to predict actions, and (iii) to integrate predicted effects of own and others' actions” (p. 70).

A more restrictive definition of joint action was first proposed by (Bratman, 1992), who discussed a very specific form of joint action, which he termed Shared Cooperative Activities. At the heart of Brattman's description of Shared Cooperative Activities, were the following tenets: (a) both actors must intend to perform the shared activity, and understand the intentions of the other, (b) be committed to the joint activity and committed to supporting the partner. In their commentary, Moreau and Candidi (2016) argued that joint actions should be constrained as those actions that involve “reciprocal and bidirectional adaptation between two agents” (p. 2). We suggest that Moreau's and Candidi (2016) definition of joint action is more restrictive and thus it aligns with Bratman's (1992) definition rather than the more all-encompassing definition of Sebanz et al. (2006).

According to Sebanz et al. (2005), “our brains might be operating somewhat like a single person constantly carrying an umbrella that is big enough for two—always ready to take others into account” (p. 1245). As such, our study aimed to assess how motor behavior is altered by the presence of a confederate. We do acknowledge the inclusion of a “passive experimenter as a confederate” (Moreau and Candidi, 2016, p. 2) in two of our conditions (pick-up and pass, and pick-up, pour and pass). In these scenarios, an active confederate would have poured a glass of water from the pitcher, and taken a drink from the mug. Nevertheless, findings demonstrated that the mere presence of a confederate does indeed alter the way objects are grasped by passer. About the way people “manipulate objects during joint actions” (p. 25), Rosenbaum et al. (2012) stated that, “handing someone a spoon in a way that reflects understanding of what the recipient will do with the spoon illustrates the way that social factors can interact with action planning” (p. 25). Moreau and Candidi (2016) claim our “set up fails to measure the dynamic encounters that adjust behavioral and cognitive processes of agents involved in joint actions” (p. 2), yet motor planning considers numerous factors, including “biomechanical efficiency and comfort, the relative importance of different costs such as the symmetry or asymmetry or bimanual movements, and considerations of others' needs” (Rosenbaum et al., 2012, p. 26).

Related work on joint action (e.g., Gonzalez et al., 2011; Ray and Welsh, 2011) has implemented similar study designs with passive recipient conditions. For example, the work of Gonzalez et al. (2011) was reviewed in our paper. Another example from Ray and Welsh (2011) required participants to pass a jug of water to examine response selection during a joint action task. Here, participants were instructed to pass a jug of water to a confederate, without any other instructions. The confederate grasped the jug and placed it on a table. In the remaining trials, the confederate always grasped the jug by the handle to pour water into a mug, regardless if this resulted in an awkward position. Participants presented the handle in both conditions; thus, supporting the idea of shared task representation in joint action planning regardless of the task intention.

While philosophical discussions within social neurosciences may have called for a shift away from “isolation paradigms” (Becchio et al., 2010) to an active interaction of more than one actor (Schilbach et al., 2013) under constrained reciprocal and bidirectional experimental setups (Sacheli et al., 2015), we would argue that accepting the restrictive (Bratman, 1992) definition of joint action is limiting. As pointed out by Milward and Carpenter (2018), neither definition is incorrect. It is important that researchers clearly articulate in their work which definition of joint action is being used. Additionally, researchers should include (a) clear statements of the roles of both actors in the shared task; and (b) whether the shared task is collaborative/cooperative or coordinated in nature.

In summary, we suggest researchers adopt the broad definition of Sebanz et al. (2006) as it provides greater flexibility in examining the construct of joint action. But equally, we should take note of Moreau's and Candidi (2016) commentary, and recognize that there may be subclasses of joint action that should be considered. It is paramount that researchers have such conversations across different disciplines, so that future work is clear about the meaning of “jointness” and how it is measured. Discussion of how collaborative, cooperative, and coordinated action fit within the joint action literature should also be considered.

Utilizing the Sebanz et al. (2006) definition allows both motor control scientists and social neuroscientists to explore a range of joint actions from slightly different perspectives that will ultimately provide a deeper understanding of how two actors work together. Too narrow a definition leaves out a range of interactive, cooperative, collaborative behaviors between agents/actors that need to be examined both from the motor control perspective and the social neuroscience perspective.

## Author contributions

SSB and PJB have made substantial, direct, and intellectual contribution to the work, and approved it for publication.

## Conflict of interest statement

The authors declare that the research was conducted in the absence of any commercial or financial relationships that could be construed as a potential conflict of interest.
